# Hemorrhagic Diathesis

**Published:** 1873-02

**Authors:** 


*Hemorrhagic Diathesis.*—The following interesting case was related of one Rufus Mitchell, of Millbridge, Massachusetts, who recently bled to death from a very slight cut:

"He was one of those unfortunate men who bleed from the slightest scratch of the skin, and many times he has lain and bled till it seemed that the blood had all run out, and then he would gradually recover. This time the cut was quite large, and he lived but a very few hours. There is something remarkable about this family, who are here termed as belonging to the bleeding family. None but the males bleed, and they are the sons of the females of the same family. For instance, this man has left children; none of them will bleed; but if the girls should have boys in their families, they will be of the bleeding kind, but the boys themselves are free and their families will be the same. I cannot explain this. I have practiced in the family for more than twenty years. During this time, a number of them have died from this cause, and others have bled, often dangerously."

A remarkable example of hemorrhagic diathesis, was related a number of years ago by Dr. Hughes, of Kentucky, which in its features closely resembles the one we stated above. The predisposition in Dr. Hughes' case, was associated with the rheumatic diathesis, and was satisfactorily traced as far back as five generations. It was confined exclusively to the male members of the different families; but the females, nevertheless, invariably transmitted it to their offspring.

An English author, Mr. Wardrop, also relates a case similar to those just given, where the most insignificant wounds bled so freely as to cause death.

